# Use of mindfulness-based interventions for parental stress in relation with ASD: A review

**DOI:** 10.1192/j.eurpsy.2022.2269

**Published:** 2022-09-01

**Authors:** M. Yesilkaya, E. Mijail Magallon Neri

**Affiliations:** 1 University of Barcelona, Clinical And Health Psychology, Barcelona, Spain; 2 University of Barcelona, Clinical Psychology And Psychobiology, Barcelona, Spain

**Keywords:** autism, parental stress, Mindfulness

## Abstract

**Introduction:**

Children with ASD may present their parents with several challenges, most notably aggressive and destructive behaviors. Parents of children with ASD typically experience more stress, depressive and anxiety symptoms than other parents. Mindfulness-based interventions are reported to be effective in reducing parental stress along with improving the challenging behaviors of their children

**Objectives:**

The current review aimed to investigate if the use of mindfulness-based interventions is beneficial to reduce parental stress when caring for children with ASD.

**Methods:**

The databases of PsycINFO and PubMED were used. The variables studied in this systematic review were Autism, Parental stress, and Mindfulness. Inclusion criteria were that all articles should be academic journals, and with a full text available in English during the last 20 years.

**Results:**

Compared to other behavioral approaches, mindfulness-based interventions showed statistically significant results in reducing the stress levels of parents as well as improving their children’s challenging behaviors.

**Conclusions:**

Most of the studies in the current review indicated that mindfulness-based interventions are beneficial in improving the stress level of parents.

**Disclosure:**

No significant relationships.

